# Impact of quality strategies on hospital outputs

**DOI:** 10.1136/qshc.2008.029439

**Published:** 2009-01-26

**Authors:** R Suñol, P Vallejo, A Thompson, M J M H Lombarts, C D Shaw, N Klazinga

**Affiliations:** 1 Avedis Donabedian Institute, Autonomous University of Barcelona, and CIBER Epidemiology and Public Health (CIBERESP), Barcelona, Spain; 2 School of Social and Political Studies, University of Edinburgh, Edinburgh, Scotland; 3 Academic Medical Center, Department of Social Medicine, University of Amsterdam, Amsterdam, the Netherlands; 4 European Society for Quality in Health Care, Limerick, Ireland

## Abstract

**Context:**

This study was part of the Methods of Assessing Response to Quality Improvement Strategies (MARQuIS) research project on patients crossing borders, a study to investigate quality improvement strategies in healthcare systems across the European Union (EU).

**Aim:**

To explore the association between the implementation of quality improvement strategies in hospitals and hospitals’ success in meeting defined quality requirements that are considered intermediate outputs of the care process.

**Methods:**

Data regarding the implementation of seven quality improvement strategies (accreditation, organisational quality management programmes, audit and internal assessment of clinical standards, patient safety systems, clinical practice guidelines, performance indicators and systems for obtaining patients’ views) and four dimensions of outputs (clinical, safety, patient-centredness and cross-border patient-centredness) were collected from 389 acute care hospitals in eight EU countries using a web-based questionnaire. In a second phase, 89 of these hospitals participated in an on-site audit by independent surveyors. Pearson correlation and linear regression models were used to explore associations and relations between quality improvement strategies and achievement of outputs.

**Results:**

Positive associations were found between six internal quality improvement strategies and hospital outputs. The quality improvement strategies could be reasonably subsumed under one latent index which explained about half of their variation. The analysis of outputs concluded that the outputs can also be considered part of a single construct. The findings indicate that the implementation of internal as well as external quality improvement strategies in hospitals has beneficial effects on the hospital outputs studied here.

**Conclusion:**

The implementation of internal quality improvement strategies as well as external assessment systems should be promoted.

This study forms part of the Methods of Assessing Response to Quality Improvement Strategies (MARQuIS) project. The objectives of the MARQuIS project are to research and compare different quality improvement (QI) policies and strategies in healthcare systems across the member states of the European Union (EU), and to consider their potential value when patients cross borders to receive healthcare. This research was intended to enable an evaluation of the need for, and development of, formal quality procedures at the EU level for healthcare services.

To identify quality strategies in Europe, quality policies were analysed, and a survey of key experts in QI from the 25 member states comprising the EU in 2005 was conducted to gather information about views and accounts of QI policies and strategies in their healthcare systems. Seven main strategies were identified^1^:

- accreditation
- organisational quality management programmes (TQMs)
- audit and internal assessment of clinical standards (internal assessment)
- patient safety systems (patient safety)
- clinical practice guidelines (clinical guidelines)
- performance indicators
- systems for obtaining patients’ views (patient’s views).

We conceptually classified the seven strategies into two groups: external strategies, which need an external model and collaboration from outside the hospital to be implemented (accreditation), and internal strategies, which are managed within the hospital and do not necessarily require external support (the other six strategies).

To study quality requirements we focused mainly on the two transversal dimensions of the PATH (Performance Assessment Tool for Quality Improvement in Hospitals) framework, and also on clinical effectiveness.^2^ A qualitative study with semi-structured interviews of patients, professionals, and financiers were carried out to explore their views regarding quality requisites, mainly related to safety and patient-centredness, when receiving or providing cross-border care.^3^ The requirements thus identified were grouped onto the four dimensions of outputs: clinical, safety, patient-centredness and cross-border patient-centredness.

Building on the information collected in previous phases of the project, the field test aimed to collect information from a sample of states (Belgium, Czech Republic, France, Ireland, Poland, Spain, the Netherlands, and the UK) to describe how hospitals have applied national quality strategies, and how far they meet the defined requirements of cross-border patients. To do so, a questionnaire was constructed including the strategies and requirements for identified outputs. Each quality strategy and output dimension was operationalised by identifying a concrete set of measures that represented the concept. The questionnaire consisted of four sections, with one section focusing on QI at the hospital level, while the other three focused on quality management of specific conditions (acute myocardial infarction, appendicitis and deliveries), selected on the basis of their frequency in cross-border care.^4^ The questionnaire was self-completed and answered by 389 European hospitals.^5^

The findings from the questionnaire allowed us to explore whether we could differentiate between hospitals according to the maturity of the QI activities they perform. To do so, we classified hospitals according to a scoring model which we called the “quality improvement (QI) maturity index”.^6^ This index was found to be useful in differentiating between hospitals according to the maturity of their QI system. To verify the results of the questionnaire and to provide supplementary data on hospital performance, an on-site audit was performed on a sample of the hospitals that participated in the questionnaire phase of the study. A purposive sample was used by randomly selecting hospitals from the two extreme quartiles of the results for the maturity index. A total of 89 hospitals were audited. Methods and results from the audit are reported elsewhere.^7^

Using the information collected with the questionnaire and the audit in a sample of hospitals from eight EU member states, this article aims to explore the association between the implementation of identified national QI strategies in hospitals and hospitals’ success in meeting the defined quality requirements that are considered intermediate outputs of the care process for three conditions: acute myocardial infarction, appendicitis and deliveries.

## MATERIAL AND METHODS

The strategy and output variables for study were defined by a concrete set of measures (items) that represented the concept. The items included in each of the strategy variables have been defined elsewhere.^5^ By way of illustration, box 1 includes the items that represented the output “safety”.

**Box 1: Measures included in the output “safety”**

- The data indicate a significant improvement in at least one element of drug safety following intervention by the committee
- Medication dispensed from the pharmacy is labelled with the name of the patient, name of the drug, concentration or strength, dosage and expiration date
- Drug storage locations are locked
- High-risk drugs are stored in a location separate from the other drugs
- Alcohol hand rub dispensers are available in patient areas
- All adult patients are identified by a bracelet
- All babies are physically identified by a bracelet
- Access to the neonatal nursery is controlled by door locks
- The data indicate a significant improvement in at least one element of safety following intervention by the committee
- Resuscitation equipment is accessible, complete, organised and functional
- All fire exits are clearly signed, useable and unobstructed

To analyse how strategies and outputs behave, quantitative indexes (summary scores) were developed for each of the seven strategies and the four outputs. After construction of the indexes, the analysis proceeded in the following steps, which will be used to describe the statistical methods and present the results:

1. analysis of QI strategies
2.
  - inter-relatedness of internal strategies (at the same organisational level)
  - associations between strategies at hospital and ward levels
3. analysis of outputs of quality requirements
4.
  - analysis of interrelatedness of outputs
5. analysis of relationship between strategies and outputs
6.
  - relationship between internal QI strategies and outputs
  - relationship between external assessment and outputs
  - relationship between the hospital’s QI maturity and outputs.

### Strategy indexes

Items were grouped conceptually, and a weighting system based on the plan-do-check-act (PDCA) cycle was used. A grade of 1 was assigned to items describing action (most mature), and a grade of 4 to items describing planning activities. We obtained eight different indexes for each of the seven strategies, covering the two data sources (questionnaire and audit), the two levels of analysis (hospital and ward), and the three wards studied (maternity, internal medicine and surgery). Box 2 illustrates the scheme of strategies:

**Box 2: Strategy indexes calculated**

Data source: questionnaire (number of hospitals included: 389)

- Level of analysis: hospital: 1 index
- Level of analysis: wards:
-
  - Maternity: 1 index
  - Internal medicine: 1 index
  - Surgery: 1 index

Data source: audit (number of hospitals included: 89)

- Level of analysis: hospital: 1 index
- Level of analysis: wards:
-
  - Maternity: 1 index
  - Internal medicine: 1 index
  - Surgery: 1 index

Internal reliability of the indexes was calculated with Cronbach α. Only in three instances was the Cronbach α low, and in these cases the items were included as standalone variables (the “accreditation” strategy at the hospital level, and the “patients’ views” strategy in medical and surgery wards), using the information from items in the audit.

### Output indexes of requirements (box 3)

Items were grouped conceptually to match each of the four categories, with no weights applied. There was only one data source for these indexes, as the outputs were studied during the audits but had not been included in the self-reported questionnaire. Since these items are managed at the ward or hospital level, the internal reliability of both levels was analysed. Theoretically, patient-centredness in general, and cross-border patient-centredness in particular, are mainly managed at the hospital level, so they would be more appropriately evaluated at this level. This was supported by the Cronbach α, which showed significantly higher values for these indexes at the hospital level than at the ward level. On the other hand, for clinical outputs, since the items included are diagnosis related and managed at the ward level, they were more appropriately evaluated at this level. The items included in safety outputs are managed at both the hospital and ward level, and given similar values for Cronbach α, there was no compelling reason to choose either level. We used the indexes at both levels, but in order to simplify the presentations of results, only the index at the hospital level is presented here.

**Box 3: Outputs indexes of requirements calculated**

Data source: audit (number of hospitals included: 89)

- Level of analysis: wards
-
  - Clinical:
  - Maternity: 1 index
  - Internal medicine: 1 index
  - Surgery: 1 index
- Level of analysis: hospital
-
  - Safety: 1 index
  - Patient-centredness: 1 index
  - Cross-border care: 1 index

### Statistical analysis

#### Analysis of QI strategies

Inter-relatedness of internal strategies (at the same organisational level) was studied by calculating the Pearson correlation coefficient between the mean scores of all six internal strategies, to test the association between pairs of strategies. Subsequently, an exploratory factor analysis was carried out to see whether the six strategies could be grouped into fewer latent dimensions. The factor extraction procedure was maximum likelihood, since severe problems with the assumption of multivariate normality were observed (i.e. |skew|>2, kurtosis >7).^8^ The type of rotation used was the oblique solution. The initial criterion to decide on the number of factors extracted was based on having an eigenvalue greater than 1.

Simple linear regression models were used to find the association of each strategy at the hospital level to that same strategy at the ward level.

### Analysis of outputs of quality requirements

Inter-relatedness of outputs was studied by calculating the Pearson correlation index and performing exploratory factor analysis, using the same method described above for improvement strategies.

### Relationship between strategies and outputs

The relationship between internal QI strategies and outputs was studied by constructing a multiple regression model to find out which strategies were related to each of the outputs. Subsequently, the Pearson correlation coefficient was calculated to see whether the latent general strategy index for each ward was related to the latent general output index.

The relationship between external assessment and output was studied by calculating the Pearson correlation coefficient to see whether each type of external assessment was related to the outputs indexes. Additionally, an “external pressure index” was created, combining nine different types of external assessment. The PDCA weighting system described above was used with each of the nine items, and the mean summary score was used to construct the index.

The relationship between the hospital’s QI maturity and output was investigated by allocating hospitals to their respective quartile based on their maturity index score,^6^ and the two extreme quartiles were tested against the output indexes using the Student t statistic for independent samples.

The higher number of hospitals that participated in the questionnaire phase (n = 389) than in the audit phase (n  =  89) allowed more powerful statistical analysis, supported by evidence of a positive agreement between the data collection methods for most of the common measures.^5^ For this reason, when we worked exclusively with the strategy indexes, we performed analyses with the information from the questionnaire, even when all the analyses were subsequently duplicated with the audit information for confirmation purposes. When analysing the relationship between strategies and outputs, we used the data from the audit because output information was only included in the audit.

## RESULTS

### Analysis of QI strategies

#### Inter-relatedness of internal strategies at the same organisational level

We aimed to explore whether the different QI strategies at any given organisational level were inter-related, meaning, for example, that hospitals with a good programme to promote patient safety would also have a good strategy for getting and using patients’ views, and for the use and analysis of performance indicators. This analysis included only the six internal QI strategies, so it excludes accreditation. Two analyses were performed: one at the hospital level and the other at the ward level. At the hospital level, the analysis of the association between pairs of strategies found that almost all QI strategies were moderately correlated,^9^ with explanation of the variation (r^2^) ranging from 6% to 32%. At the ward level, low to moderate correlation was found between most strategies, with only one exception in the maternity ward (clinical guidelines with patients’ views), and two exceptions in internal medicine wards (clinical guidelines with patients’ views and clinical guidelines with performance indicators), where no statistically significant correlation was found. The analysis was replicated with data from the audit for validation purposes, and consistent results were obtained: at the hospital level explanation (r^2^) ranged from about 5% to about 35% of the variation between pairs of strategies, and at the ward level significant correlations were found for all strategies, with the same exception as mentioned above. Based on these results, there is evidence of a positive association between strategies, indicating that hospitals are using all these strategies together in relatively similar proportions.

Given this level of inter-relatedness, we used exploratory factor analysis to see whether the six internal QI strategies could be grouped into fewer latent dimensions, separately at the hospital and ward levels. At both levels of analysis we found that the six quality strategies could be reasonably subsumed into one latent index. By way of illustration, table 1 shows the dimension identified at the hospital level, which explained 47% of the variation with a Cronbach α of 0.724. All the strategies showed similar loading, indicating that the latent index explained similar proportions of the variation for each strategy. In our analysis at the ward level, we identified one dimension for each of the wards, with 42% of the variability in maternity and 40% in both internal medicine and surgery wards. The analysis was replicated with data from the audit for validation purposes, and consistent results were obtained: at the hospital level, one dimension explained 51% of the variation, and the results at the ward level were almost exactly the same. Therefore, the latent general strategy index could be used as a proxy for each strategy index in combination in the subsequent analyses.

**Table 1:** Results of inter-relatedness of strategies (factor analysis)

Data source: questionnaire (n = 389)	
Level of analysis: hospital	
Strategy indexes at hospital level	Loading weights
Performance indicators	0.717
TQM	0.706
Patient safety	0.695
Clinical guidelines	0.692
Internal assessment	0.656
Patient views	0.652
Total variation explained	47%
Cronbach α	0.724

### Associations between strategies at hospital and ward levels

The association between each strategy at the hospital level and that same strategy at the ward level was assessed to see whether the development of any specific strategy at the hospital level was related to a more developed stage of that same strategy at the ward level. The results of the analysis showed that each strategy at the hospital level was associated with the development of that same strategy at the ward level, with only one exception (clinical guidelines for internal medicine and surgery wards). The explanation (r^2^) ranged from only 1% for patient safety in surgery wards to 48% for TQM in maternity wards. To provide a clearer understanding of the strength of the association, table 2 shows the magnitude of the change in the strategies at the ward level per unit of that same strategy at the hospital level.

**Table 2:** Associations between strategies at the hospital and ward levels (simple linear regression models)

Data source: questionnaire (n = 389)						
Level of analysis: hospital and wards						
Variables (at hospital/ward level)	Maternity		Internal medicine		Surgery	
	r^2^	Change in explanatory strategies per unit of hospital strategy	r^2^	Change in explanatory strategies per unit of hospital strategy	r^2^	Change in explanatory strategies per unit of hospital strategy
TQM	48	1.19 (1.04 to 1.34)	28	0.49 (0.40 to 0.58)	45	1.09 (0.95 to 1.23)
Internal assessment	26	0.57 (0.44 to 0.70)	25	0.46 (0.36 to 0.56)	26	0.59 (0.47 to 0.71)
Patient safety	7	0.42 (0.24 to 0.60)	11	0.44 (0.30 to 0.58)	1	0.49 (0.33 to 0.66)
Clinical guidelines	4	0.33 (0.14 to 0.52)	NS		NS	
Performance indicators	3	0.13 (0.03 to 0.23)	4	0.21 (0.08 to 0.34)	4	0.23 (0.09 to 0.38)
Patients’ views	7	0.25 (0.13 to 0.38)	2	0.13 (0.04 to 0.22)	2	0.14 (0.04 to 0.24)

NS, not significant; TQM, quality management programme.

### Analysis of output quality requirements

We aimed to explore whether the different output indexes were interrelated, meaning, for example, that hospitals with good intermediate results (outputs) in safety would also have good intermediate results in clinical or cross-border patient-centredness. Moderate to strong correlation was found between all pairs of output indexes, with patient-centredness and safety being the most strongly related (r = 0.561). Explanation of the variation (r^2^) ranged from about 6% to 33% of the variability between pairs of outputs. This finding suggests that hospitals tend to perform in different areas at a similar developmental level.

Using the logic outlined earlier, we carried out an exploratory factor analysis to see whether the outputs could be grouped into fewer latent dimensions. We found that the output indexes could be considered part of a single construct, with explanation of the variation exceeding 50% (see table 3). The latent general output index explained each of the output indexes in roughly equal measure, providing a proxy for each output index in combination.

**Table 3:** Results of inter-relatedness of outputs (factor analysis)

Data source: audit (n = 89)	
Level of analysis: hospital and wards	
Strategy indexes at hospital level	Loading weights
Clinical maternity (ward level)	0.617
Clinical medical (ward level)	0.766
Clinical surgery (ward level)	0.578
Safety (hospital level)	0.761
Patient-centredness (hospital level)	0.778
Cross-border patient-centredness (hospital level)	0.740
Total variation explained	50.6%
Cronbach α	0.754

### Relationship between strategies and outputs

#### Relationship between internal QI strategies and outputs

The six internal QI strategies were included in the model, and the analysis was carried out at the ward level only. The strategies at the hospital level were not analysed against the outputs due to this information being used as a selection criterion for the hospital audits. Hospital ownership and hospital type (teaching) were included in the models as potential confounding effects. The results are presented in table 4. In all wards studied we found several strategies to be associated with each of the output indexes. Explanation (r^2^) ranged from 20% to 71%. Patient safety, performance indicators and clinical guidelines were the three strategies that were most frequently associated with the outputs. At the other extreme, the TQM strategy (at the ward level) accounted for no variation in the results. There was no evidence of an effect of hospital ownership or hospital type on any output measure.

**Table 4:** Results of relation between strategy indexes and output indexes (multiple regression model)

Data source: audit (n = 89)						
Level of analysis: wards						
Response variables: output measures	Maternity		Internal medicine		Surgery	
	Explanatory strategies in descending rank order	r^2^ (%)	Explanatory strategies in descending rank order	r^2^ (%)	Explanatory strategies in descending rank order	r^2^ (%)
Clinical	Performance indicators	52	Performance indicators	71	Performance indicators	63
	Internal assessment		Clinical guidelines		Clinical guidelines	
						
Safety	Patient safety	47	Patient safety	33	Patient safety	40
	Patient views				Clinical guidelines	
						
Patient-centredness	Patient views	32	Performance indicators	30	Performance indicators	28
	Patient safety		Patient safety		Clinical guidelines	
						
Cross-border patient-centredness	Patient safety	30	Patient safety	22	Patient safety	20
	TQM		Performance indicators			

TQM, quality management programme.

To further explore the relationship between the implementation of QI strategies and the positive results for outputs, the latent general strategy index for each of the three wards studied was related to the latent general output index. The correlations were high and significant (p<0.01) for all three wards, with the Pearson correlation coefficient ranging from 0.762 in maternity wards (n = 68) to 0.756 in surgery wards (n = 66).

### Relationship between external assessment and outputs

In addition to testing each type of external assessment system, we created an “external pressure index” which included variables that explored whether the hospital had any type of external assessment (such as certification or accreditation), whether QI activities involving external pressure were used in the organisation (such as peer review or inspections), whether the hospital had adopted the European Foundation for Quality Management (EFQM) model to implement quality strategies, and whether the laboratories (clinical chemistry, pathology, microbiology, pharmacy, diagnostic radiology) were periodically audited by an accreditation or certification institute. Table 5 summarises the results of this analysis.

**Table 5:** Results of relation between external assessment and output indexes (Pearson correlation coefficient)

Data source: audit (n = 89)						
Level of analysis: wards						
	Clinical			Safety	Patient-centredness	Cross-border patient-centredness
	Maternity	Surgery	Medical			
Government accreditation	NS	NS	p = 0.060	p = 0.009	NS	NS
Voluntary accreditation	NS	NS	p = 0.019	p<0.001	NS	p = 0.020
Teaching accreditation	NS	NS	p = 0.002	NS	NS	NS
ISO	NS	NS	NS	NS	p = 0.020	p = 0.034
External pressure index	NS	NS	NS	p = 0.007	p = 0.007	p = 0.002

ISO, International Organization for Standardization; NS, not significant.

Teaching accreditation showed just one significant association with clinical outputs for the internal medicine ward. Government accreditation was associated with safety outputs and clinical outputs of the internal medicine ward only. Voluntary accreditation showed the same associations as government accreditation, and also showed positive results for cross-border patient-centredness. International Organization for Standardization (ISO) certification was positively associated with patient-centredness and cross-border patient-centredness. The external pressure index was associated with all hospital outputs, but there was insufficient evidence of a link with ward-level outputs.

### Relationship between the hospital’s QI maturity and output

We explored whether maturity of the hospitals in QI (based on their score on the QI maturity index^6^ was related to any output indexes. As table 6 shows, hospitals with a higher maturity score (lower scores represented greater maturity) also had better global output scores (Student t = –3.336, p<0.001).

**Table 6:** Results of relation between maturity index and output indexes (Student t statistic for independent samples)

	Maturity index classification (mean overall score in classification audit)	Number	Mean (SD)	p Value
Global output	Most mature*	32	–0.399 (1.030)	<0.001
	Least mature	35	0.366 (0.84)	

*Lower values represent greater maturity.

## DISCUSSION

The study provides useful information about the behaviour of hospital QI strategies and outputs. The association between the implementation of strategies with hospital outputs supports their potential value in understanding and improving healthcare quality. One of the limitations of this study is that it only includes the intermediary outputs of the care delivery process, but not outcomes. The characteristics of the study, especially the differences in the information available from participating countries, did not allow the inclusion of outcomes. Furthermore, being an observational, non-randomised study, the association between QI strategies and hospitals’ compliance with output requirements makes proof of causality very difficult as it may be confounded by other factors.

Our analysis of QI strategies in hospitals found that all strategies are moderately intercorrelated. This allowed us to reduce data into unitary latent indexes. The fact that all dimensions were inter-related has relevant implications for safety. The recent focus on patient safety promotion and development had raised the question of whether this is part of QI, or is a completely different field. Our results reinforce the conceptualisation of safety as one dimension of quality, a finding that is in line with other recent studies.^10^ The inter-relation of strategies lends support to the use of a QI maturity index that combines several strategies and facilitates a comprehensive assessment and evaluation of the development of QI in hospitals. The positive association between hospitals’ scores on the maturity index and their results for hospital outputs provided additional support in this direction.

We also identified an association between the implementation of strategies at the hospital and the ward level, although in some cases the association was weak. This seems to indicate that the interface between a hospital and its wards is to some extent influenced through the implementation of QI strategies, as opposed to the vision of the hospital as a group of independently managed “fiefdoms”.

The analysis of outputs concluded that the outputs also can be considered part of a single construct. This result suggests that hospitals may be considered more mature or less mature in terms of the achievement of outputs, but this hypothesis needs to be further tested with more specifically focused research.

In our assessment of the association between the implementation of QI strategies and hospital outputs, we found several strategies to be linked. Patient safety systems, performance indicators, and clinical guidelines are the strategies most clearly associated with the outputs, but all strategies influence different types of output. The correlations between the latent general strategy index and the latent general output indexes were high for all three wards studied. This lends support to the possibility of a causal relationship between strategies and outputs, albeit requiring further research. Based on these results, the implementation of QI strategies in hospitals seems to have an effect in promoting positive change in organisations. Implementation of the strategies included in this study may be a good starting point, but hospitals could also explore other strategies. Several studies that explored relations between QI activities and outputs or outcomes of care have recently been published. These studies are in line with the present report, although most of them were focused on specific areas such as acute myocardial infarction,^11^ ^12^ acute coronary syndromes,^13^ cancer care^14^ and general surgery.^15^ The proliferation of such studies reflects the need to understand the consequences of implementing QI initiatives, and gains in our understanding of these consequences will allow further assessment of the effectiveness and accountability of specific QI practices.

Voluntary accreditation, teaching accreditation and ISO certification are all associated with some of the outputs studied here, and the external pressure index is positively associated with all hospital outputs in the present analysis. Therefore, the external assessment of hospitals seems to be a positive strategy. This is an important finding because a recent review of accreditation studies found that accreditation programmes promote change in health organisations, but that the changes are related to standardising the organisation and decision-making processes for care rather than producing care outcomes. The finding of associations between external assessments and outputs is therefore a preliminary positive finding that needs to be further explored.^16^ Since all external assessment methods had some positive influence on the results, there is no reason to promote any particular one; moreover, the type of external pressure may differ according to the characteristics of the organisation, its goals and context. It is important to note that more than 80% of the hospitals included in the study had undergone some type of external assessment either of the whole hospital or parts of it, so it seems that European hospitals are moving in a positive direction.

We would like to stress that we did not find any evidence of an effect of hospital ownership or hospital teaching status on any output measures. These two variables were included in the study because they are considered a proxy for other important characteristics of hospitals in organisational studies.^17^ ^18^ On the basis of these findings, there is no reason to consider that QI strategies will differ depending on a hospital’s teaching or ownership status.

In conclusion, the evidence indicates that implementation of internal as well as external QI strategies in hospitals has beneficial effects on the hospital outputs studied here.
